# Acute subanesthetic ketamine-induced effects on the mismatch negativity and their relationship to early and sustained treatment response in major depressive disorder

**DOI:** 10.1177/02698811251319456

**Published:** 2025-02-26

**Authors:** Sara de la Salle, Jennifer L Phillips, Pierre Blier, Verner Knott

**Affiliations:** 1 University of Ottawa Institute of Mental Health Research at the Royal, Ottawa, ON, Canada; 2 School of Psychology, University of Ottawa, Ottawa, ON, Canada; 3 Department of Psychiatry, University of Ottawa, Ottawa, ON, Canada; 4 Department of Biochemistry, Microbiology and Immunology, University of Ottawa, Ottawa, ON, Canada; 5 Department of Cellular and Molecular Medicine, University of Ottawa, Ottawa, ON, Canada

**Keywords:** Major depressive disorder, event-related oscillations, ketamine, midazolam, mismatch negativity, cortical source density, treatment response

## Abstract

**Background:**

A sub-anesthetic dose of ketamine, an N-methyl-D-aspartate receptor (NMDAR) antagonist, produces robust antidepressant effects in treatment-resistant major depressive disorder (MDD). The mismatch negativity (MMN) is reliant on glutamatergic neurotransmission and reduced by NMDAR antagonists. The MMN may characterise the neural mechanisms underlying ketamine’s effects.

**Aims:**

This study examined the acute effects of ketamine and midazolam on the MMN and its relationship to early and sustained decreases in depressive symptoms.

**Methods:**

Treatment-resistant MDD patients (*N* = 24), enrolled in a multi-phase clinical ketamine trial, received two intravenous infusions within an initial double-blind crossover phase: ketamine (0.5 mg/kg) and midazolam (30 μg/kg). Three recordings were carried out per session (pre-, immediately post- and 2 h post-infusion). Peak MMN amplitude (μV), latency (ms), theta event-related oscillations (EROs), theta phase locking factor (PLF) and source-localised MMN generator activity were assessed. Relationships between changes in MMN indices and early (Phase 1: double-blind, cross-over phase) and sustained (Phases 2, 3: open-label repeated and maintenance phases, respectively) changes in depressive symptoms (Montgomery-Åsberg Depression Rating Scale score) were examined.

**Results:**

Ketamine reduced frontal MMN amplitudes, theta ERO immediately post- and 2 h post-infusion and source-localised peak MMN frontal generator activity. Select baseline and ketamine-induced MMN decreases correlated and predicted greater early (left frontal MMN decreases in amplitude and theta ERO, baseline left PLF) and sustained (baseline left PLF, right inferior temporal activity) symptom reductions.

**Conclusions:**

Acute NMDARs blockade reduced frontal MMN, with larger MMN reductions predicting greater symptom improvement. The MMN may serve as a non-invasive biomarker predicting antidepressant response to glutamatergic agents.

## Background

Despite decades of research and numerous pharmacotherapeutic options, the pathophysiology of major depressive disorder (MDD) remains poorly understood. Current treatments primarily target the monoamine systems (serotonin, noradrenaline, dopamine), whose purported underactivity is responsible for depressive symptoms (Boku et al., 2018). Pharmacological agents which increase monoamine levels (i.e. drugs for depression: tricyclic antidepressants, selective serotonin reuptake inhibitors, serotonin–norepinephrine reuptake inhibitor) are commonly prescribed for MDD. However, up to two-thirds of treatment-seeking patients do not achieve symptom relief and are classified as having treatment-resistant (TR) depression (Kennedy et al., 2001; Rush et al., 2006).

One promising area of investigation involves the glutamate system (Murrough et al., 2017). Research has indicated that alterations in the concentrations and activity of glutamate, the primary excitatory neurotransmitter and gamma-aminobutyric acid (GABA), the primary inhibitory neurotransmitter, have been observed in MDD populations, suggesting that excitatory/inhibitory signalling dysfunction may contribute to the disorder (Lener et al., 2017). Glutamate plays a crucial role in regulating synaptic plasticity and is integral to mood, cognition, learning and reward (Murrough et al., 2017).

The N-methyl-D-aspartate receptor (NMDAR) antagonist ketamine has received considerable focus within human and animal research in the past 2 decades (Wei et al., 2020). While the exact mechanism has not been completely elucidated, current understanding suggests that ketamine exerts its antidepressant effects through increased glutamate neurotransmission in the prefrontal cortex (PFC) through NMDAR blockade and α-Amino-3-hydroxy-5-methyl-4 isoxazolepropionic acid (AMPA) activation, leading to an increase in synaptic plasticity and neurotrophic signalling through downstream molecular cascades (Lener et al., 2017; Murrough et al., 2017). NMDA receptor antagonism in the PFC paradoxically increases glutamatergic transmission through several mechanisms: selectively suppressing GABAergic interneuron activity, enhancement of presynaptic glutamate release and activation of non-NMDA glutamate receptors (e.g. AMPA receptors; Egunlusi and Joubert, 2024; Homayoun and Moghaddam, 2007). This process is further complicated by increased dopamine release, which interacts with the glutamatergic system through D1 receptor activation (Wu et al., 2021a, 2021b). The temporal dynamics of these effects are complex, indicating that the timing and frequency of ketamine administration can markedly influence both treatment efficacy and neuroplastic changes (Wu et al., 2021a), with important for optimizing ketamine’s therapeutic potential in treating MDD.

Numerous independent studies and meta-analyses have consistently shown that a single sub-anesthetic dose of ketamine is able to produce rapid antidepressant effects in individuals with MDD (Caddy et al., 2015; Coyle and Laws, 2015; Fond et al., 2014). However, these effects are typically transient, peaking within 24 h, with depressive symptoms often returning within approximately 1-week post-infusion (Kishimoto et al., 2016). Most placebo-controlled trials have used saline, however, the benzodiazepine midazolam used as an ‘active’ placebo was found to be superior in terms of blind integrity (Wilkinson et al., 2019). Recent studies indicated that repeated ketamine infusions can prolong antidepressant effects (Aan Het Rot et al., 2010; Cusin et al., 2017; Murrough et al., 2013; Shiroma et al., 2014; Singh et al., 2016; Vande Voort et al., 2019; Wilkinson et al., 2018). For instance, Phillips et al. (2019) found that repeated infusions administered thrice weekly for 2 weeks resulted in cumulative and sustained responses maintained in responders during maintenance infusions (once weekly, for a month).

Understanding how ketamine alters neural functioning may enhance our understanding of its antidepressant effects. The auditory mismatch negativity (MMN) event-related potential is a widely studied early electroencephalographic (EEG) measure primarily modulated by NMDA receptor signalling (Rosburg and Kreitschmann-Andermahr, 2016) and has been suggested as a neurophysiological proxy of target engagement for NMDAR-associated cortical plasticity (Kantrowitz et al., 2018). The MMN, a frontocentral negative deflection peaking approximately 120–250 milliseconds (ms) post-stimulus, reflects prediction error in sensory processing (Fitzgerald and Todd, 2020) based on regularity violation rather than solely auditory ‘echoic’ sensory memory (Näätänen, 1990). It occurs when an infrequent stimulus deviates from expectations set by previous stimuli (Winkler, 2007), demonstrating the brain’s capacity to detect differences between expected and actual input. This phenomenon aligns closely with predictive coding, which suggests that the brain constantly formulates predictions about incoming sensory data and revises these predictions as new information arrives. As such, the MMN is thought to reflect the degree of alignment between top-down and bottom-up systems, where a stronger response signals a larger prediction error, prompting a greater reallocation of processing resources (Fitzgerald and Todd, 2020; Murphy et al., 2022).

The MMN is generated by various brain regions, including the bilateral superior temporal lobe, anterior cingulate gyrus and right inferior temporal lobe (Waberski et al., 2001). Its temporal subcomponent arises from pre-perceptual deviance detection, followed by involuntary switching of attention to the auditory deviance by frontal regions, producing the frontal MMN subcomponent (Rinne et al., 2000). Preceding the MMN is the N1 ERP, which reflects early sensory processing and is sensitive to attention and stimulus characteristics, showing adaptive patterns with repeated stimuli (Hillyard et al., 1973; Zhang et al., 2011). The N1 represents the immediate auditory detection response, while the MMN reflects the detection of unexpected changes in auditory patterns, both of which may be altered in MDD (Key et al., 2023; Tseng et al., 2021) and sensitive to NMDA antagonists (Wang et al., 2024). While not extensively examined in MDD research, certain studies have reported reduced MMNs (i.e. decreased amplitude, Qiao et al., 2013; Takei et al., 2009) as well as MMN increases (Bissonnette et al., 2020; He et al., 2010; Kähkönen et al., 2007; Restuccia et al., 2016) and longer latencies (Bissonnette et al., 2020; Qiao et al., 2013). A recent study also found a relationship between MMN amplitude and functional outcomes in MDD patients (Kim et al., 2020), suggesting that findings of reduced amplitudes may reflect pathological NMDAR functioning, which subsequently leads to impaired functioning.

While MMN analyses typically focus on peak amplitude (microvolts (μV)) and latency (ms) in the time domain, underlying phase-locked frequency-specific event-related oscillations (ERO) and measures of time-varying phase locking (PLF) have been found to influence these measures. Measured in the time-frequency domain, ERO and PLF can provide sensitive and detailed descriptions of brain dynamics that underlie the MMN (Kaser et al., 2013). Theta EROs are intricately related to the MMN despite differing time scales. Both share neurophysiological mechanisms involving NMDA receptors and similar cortical generators (Javitt et al., 2018). Theta oscillations are thought to support MMN generation by coordinating neural activity for change detection and information processing (Ko et al., 2012). Increases in theta ERO and phase alignment for deviant stimuli are related to the frontal MMN subcomponent, while theta phase resetting without ERO modulation has been observed with the temporal component (Fuentemilla et al., 2008). Understanding their connection provides deeper insights into the functional, spatial and temporal dynamics of auditory deviance detection.

NMDA antagonists have been shown to alter the MMN in both human (Guo et al., 2024) and animal studies (Gil-Da-Costa et al., 2013). Ketamine reduces MMN amplitudes and increases latencies, specifically affecting responses to frequency and duration deviants (Guo et al., 2024; Kenemans and Kähkönen, 2011; Rosburg and Kreitschmann-Andermahr, 2016). Correlational findings have also linked smaller pre-ketamine MMNs to more significant ketamine effects (Umbricht et al., 2002). Glycine, an NMDA enhancer, selectively reduces frontal MMN but not temporal MMN (Leung et al., 2008), suggesting region-specific MMN effects, while another NMDA antagonist, memantine, increases MMN amplitude in EEG but not in magnetoencephalography, indicating frontal, not temporal, MMN impacts (Korostenskaja et al., 2007). As theta oscillations and phase-locking underlie MMN generation, NMDAR antagonists have also been found to alter both spontaneous (Hunt and Kasicki, 2013) and auditory evoked theta activity (de la Salle et al., 2019; Lee et al., 2018).

The administration of the MMN requires no behavioural response or attention (a cognitive domain that is markedly altered with ketamine) as it captures pre-attentive detection processes. Combined with its reliance on NMDAR functioning and its purported relation to synaptic plasticity, the MMN has emerged as an efficient, feasible, non-invasive neurobiomarker for monitoring target engagement linked to the activity of NMDA receptors (Kantrowitz et al., 2018) and for predicting antidepressant response to glutamatergic agents (Murphy et al., 2022). A recent study (Sumner et al., 2020) examined the use of a roving MMN paradigm to investigate the downstream effects of ketamine on predictive coding and short-term plasticity in a sample of patients with MDD receiving a sub-anesthetic dose of ketamine (0.44 mg/kg). The authors selected to examine these effects 3–4 h post-infusion in order to assess improvements in depressive symptoms; they noted increases in MMN amplitudes at this time point, interpreting this increase as a restoration of sensitivity to prediction errors found in MDD. However, these findings do not probe the early ketamine effects on the brain (i.e. immediately post-infusion or 2 h post-infusion when decreases in depression symptoms may begin to be observed) and do not assess the longer-term implications of this change in MMN on depression response.

This ERP study was a component of a larger clinical trial (NCT01945047) aimed at enhancing and prolonging the antidepressant effect of ketamine through repeated infusions (Phillips et al., 2019). The objective was to examine the utility of MMN measures (amplitude, latency, ERO, PLF, source-localised peak MMN generator activity) in characterising the acute electrocortical effects of midazolam and a sub-anesthetic dose of ketamine in TR MDD and to examine their relationship to early and sustained changes in depressive symptoms with repeated ketamine treatments. A single deviant type (longer duration) that has been consistently found to elicit diminished MMNs in patients with MDD (Tseng et al., 2021) and to be responsive to ketamine administration was employed (de la Salle et al., 2019; Gunduz-Bruce et al., 2012; Heekeren et al., 2008; Kreitschmann-Andermahr et al., 2001; Mathalon et al., 2014; Roser et al., 2011; Umbricht et al., 2000). In addition to measuring the amplitude and latency of MMN, its underlying oscillatory dynamics were assessed by time-frequency analysis to yield phase-locked frequency-specific EROs and time-varying PLF measures (Kaser et al., 2013), which in the theta frequency (4–7 Hz) range, differentially contribute to the generation of the temporal and frontal MMN components (Fuentemilla et al., 2008). Furthermore, the activity of underlying frontal and temporal intra-cortical generators contributing to surface MMN recordings was assessed with exact low-resolution brain electromagnetic tomography (Pascual-Marqui et al., 2011). Consistent with previous healthy control studies measuring acute changes in MMN, we expected initial acute decreases in MMN, measures of theta (ERO and PLF) and activity of frontal and temporal MMN generators, followed by a potential increase at 2 h post-ketamine infusion. We also expected to observe relations between changes in MMN measures and early and sustained antidepressant treatment response.

## Methods

### Participants

The participant sample is described in de la Salle et al. (2022). Briefly, 24 outpatients (males and females, aged 18–65 years) with TR MDD participated in an add-on electrophysiological arm of a larger single-centre randomised controlled trial (Phillips et al., 2019). Inclusion criteria were: (i) a primary Axis I diagnosis of MDD, single or recurrent and without psychotic features, confirmed using the Mini-International Neuropsychiatric Interview (Sheehan, 1998) and assessed using criteria from the Diagnostic and Statistical Manual of Mental Disorders, 4th edition (DSM-IV-TR, American Psychiatric Association, 2000), (ii) treatment-resistance, defined as failure to adequately respond to at least two trials of medications for depression (of different pharmacological classes) and two augmentation strategies for a minimum of 6 weeks during the current depressive episode (using the Antidepressant Treatment History Form, Sackeim, 2001). Participants maintained stable dosages of psychotropic medications for at least 6 weeks prior to treatment, with no changes allowed during the trial (see de la Salle et al., 2022, Supplemental material). A Montgomery Åsberg Depression Rating Scale (MADRS; Montgomery and Åsberg, 1979) total score of ⩾25 was required at screening and randomisation, with no more than 20% improvement between these visits. Exclusion criteria included: (i) current or past problematic substance use or dependence (defined by DSM-IV-TR or positive urine screen), (ii) psychotic symptoms, (iii) history of mania or hypomania, (iv) body mass index ⩾ 35 and (v) unstable medical conditions determined by physical examination, vital signs, weight, electrocardiogram, blood tests and urinalysis (including pregnancy testing for females). Informed consent was obtained from all participants for the clinical trial and add-on EEG arm. The study was approved by the Research Ethics Boards of the Royal Ottawa Health Care Group and was conducted in accordance with the Tri-Council Policy Statement on Ethical Conduct for Research Involving Humans.

### Study design

The clinical trial (NC101945047) comprised three phases. Phase 1 utilised a randomised, double-blind crossover design, with participants assigned to receive either ketamine (KET) or an active placebo (midazolam, MID) for the first infusion while keeping participants and immediate study staff blind to the treatment. To receive the second infusion, participants had to return to 80% of their baseline MADRS scores, with a minimum of 7 days between sessions. The same criteria applied for progression to Phase 2.

In Phase 2, all participants received acute repeated administration of six open-label ketamine infusions, administered thrice weekly over 2 weeks. Only those who met the antidepressant response criteria (i.e. ⩾50% decrease in MADRS total score from baseline (prior to first infusion in Phase 1) to the end of Phase 2) moved to Phase 3, the maintenance phase, which consisted of four open-label ketamine infusions, given once weekly over 4 weeks.

During each session of Phase 1, EEG recordings were taken before, immediately after and 2 h post-infusion.

### Drug administration

Ketamine hydrochloride (Ketalar^®^, ERFA Canada Inc., Montreal, QC; 0.5 mg/kg, diluted in 0.9% saline) was administered throughout all three study phases. Midazolam (30 μg/kg diluted in saline) was administered once during Phase 1. Both medications were delivered by intravenous pump over 40 min by a study physician and research nurse in an outpatient setting. Vital signs (blood pressure, pulse, oxygen saturation) were monitored throughout and after the infusion to ensure they returned to pre-infusion levels. Participants were required to abstain from benzodiazepines the day before (Frye et al., 2015) and from grapefruit juice on the day of infusion (Peltoniemi et al., 2012).

### EEG acquisition

**Figure 1.**
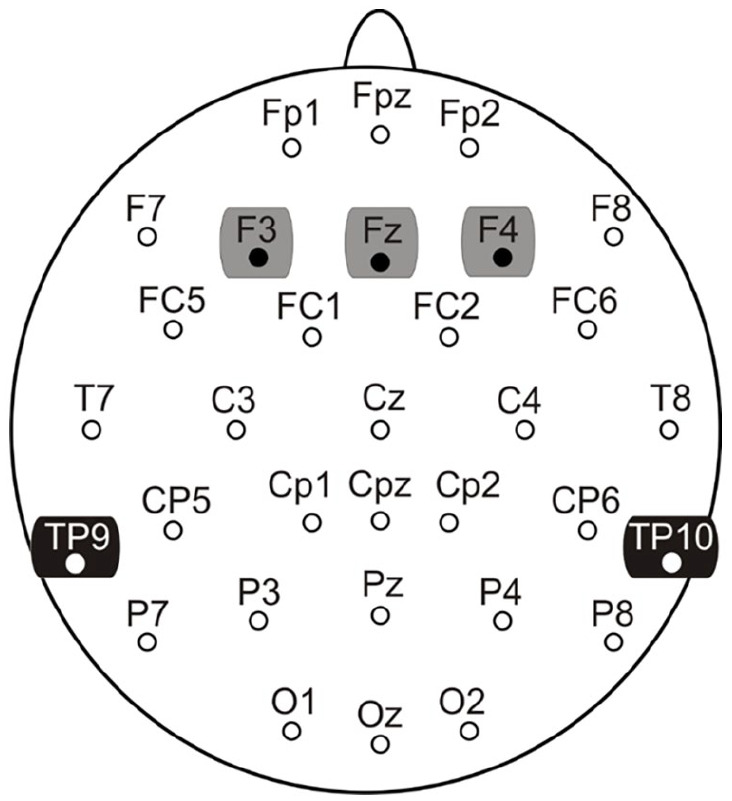
Electrode recording montage and electrode groupings (frontal = grey, mastoids = black) for analysis. Highlighted electrodes indicate sites of mismatch negativity (MMN) measurement.

Electrophysiological recordings were performed according to standard pharmaco-EEG procedures (Jobert et al., 2012) using a Brain Vision^®^ Quickamp amplifier and Brain Vision Recorder^®^ (Brain Products, Germany), with amplifier bandpass filters and sampling rate set to 0.1–100 Hz and 500 Hz, respectively. Electrode impedances were maintained below 5 kΩ. The montage included 32 Ag^+^/Ag^+^Cl^−^ passive electrodes (placed according to the 10–20 international EEG system; Figure 1) and an electrode placed on the nose served as a reference. A mid-forehead electrode (AFz) served as the ground. The electro-oculographic activity was recorded from bipolar electrodes placed on the supra- and sub-orbital ridges of the right eye and on the external canthus of both eyes to record vertical (VEOG) and horizontal (HEOG).

### Paradigm

The MMN paradigm was administered following 3 min of eyes closed resting EEG. Participants viewed a silent subtitled nature film (Madagascar, BBC, 2011). Auditory tonal stimuli (70 dB sound pressure level) were presented binaurally through noise-cancelling headphones. The sequence consisted of 1350 high probability (*p* = 0.9) standard stimuli (1000 Hz, 50 ms), beginning with a series of 15 standard stimuli and randomly intermixed with 150 low probability (*p* = 0.1) longer duration deviant stimuli (1000 Hz, 100 ms), with a 500 ms fixed stimulus onset asynchrony.

### MMN analysis

Brain Vision^®^ Analyzer Version 2.2 software (Brain Products, Munich, Germany) was used for offline ERP and wavelet processing. Raw data was re-referenced to the nose, bandpass filtered from 1 to 20 Hz (24 dB/oct; (Duncan et al., 2009), ocular-corrected (Gratton et al., 1983) and segmented (−100 to 400 ms) separately for the standard and deviant stimuli. The segments were inspected semi-automatically for artifacts (voltages ± 75 µV, faulty channels, drift) and baseline corrected (relative to the pre-stimulus segment). Difference waveforms, derived by digital point-by-point subtraction of the standard stimuli from the deviant stimuli (Näätänen et al., 2007), were computed for each electrode site. The MMN was quantified (µV) as the most negative peak (±5 ms) within 120–250 ms at the frontal electrodes (F3, Fz, F4) and as the most positive peak at the temporal (mastoid) electrode sites (TP9, TP10) in order to assess both MMN subcomponents. The obligatory N1 ERP elicited by the standard stimuli was defined as the most negative peak between 90 and 120 ms at the frontal midline electrode (Fz). Latencies (ms) for the MMN and the N1 were derived from the frontal midline site (Fz).

EROs (µV) were computed for averaged time-frequency epochs using a complex Morlet wavelet with a constant (*c* = σ_*f*_/*f*, where σ_*f*_ = the standard deviation of the centre frequency (*f*)) value of 5 over the range of 1–20 Hz, 1 Hz per frequency step (Hall et al., 2011). PLF values were computed using the PLF solution following wavelet transformations for each epoch and subtracting the standard epochs from the deviants. PLF was calculated as 1 minus the circular variance of phases, with ranges from 0 (random distribution of phases) to 1 (perfect phase locking, Tallon-Baudry et al., 1996). Frontal (F3, F4, Fz) theta (4–8 Hz) ERO and PLF were exported for ±50 ms around peak MMN amplitude. ERO values were then ln-transformed to ensure that the data were normally distributed for statistical analysis.

### Source-localised activity for MMN generator regions of interest (ROIs)

**Figure 2.**
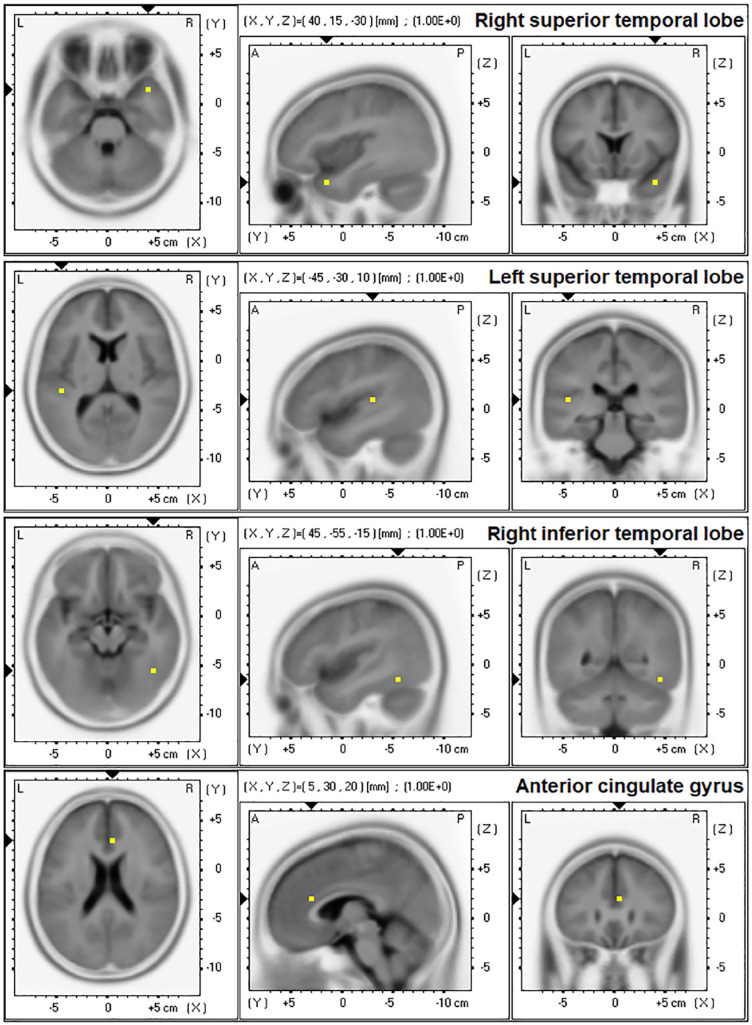
Mismatch negativity (MMN) temporal and frontal generator regions of interest (ROI) as measured with exact low-resolution electromagnetic tomography (eLORETA) and as defined by Waberski et al. (2001).

Source-localised current density (A/m²) was measured at peak MMN activity (based on ERP grand averages) from four previously defined ROIs representing four identified generators of the MMN (Heekeren et al., 2008; Waberski et al., 2001). This was performed with exact low-resolution electromagnetic tomography (version 2081104; Pascual-Marqui et al., 2011). eLORETA is a weighted minimum non-linear inverse solution method applied to EEG recordings for computation of the three-dimensional distribution of electric cortical activity with zero location error (Pascual-Marqui et al., 2011). The ROIs (Figure 2) were based on eLORETA-defined Brodmann Areas (BA) and included the four generators defined by Waberski et al. (2001): right superior temporal lobe (rSTL, BA38), left superior temporal lobe (lSTL, BA41), right inferior temporal lobe (rITL, BA37) and the anterior cingulate cortical gyrus (ACC, BA32). Current density data from a single centroid representative voxel of each BA (the voxel closest to the centre of the BA mass, which is an excellent representation of the corresponding BA) were extracted for further analysis. These ROIs were defined based on the Montreal Neurologic Institute average MRI brain (MNI 152) (Mazziotta et al., 2001) consisting of 6239 voxels (5 × 5 × 5 mm³/voxel) and restricted to cortical gray matter/hippocampus. This method has been cross-validated with functional and structural MRI, PET and intracranial recordings (Mulert et al., 2004; Pizzagalli et al., 2004; Seeck et al., 1998; Vitacco et al., 2002; Worrell et al., 2000).

### Clinical outcomes and psychoactive symptom assessment

Clinical outcome measures for the primary study are presented in (Phillips, Norris and Talbot, 2019). For this smaller sample of participants who completed the EEG component of Phase 1, mean (±SD) MADRS total scores are presented. Baseline measures were derived from the first session (regardless of drug). Comorbid disorders were assessed using the MINI at screening.

During Phase 1, the psychoactive effects associated with subanesthetic ketamine were measured with the Brief Psychiatric Rating Scale–Positive Symptoms subscale (BPRS-P; (Grunebaum et al., 2018; Overall and Gorham, 1962), which assesses conceptual disorganization, mannerisms and posturing, grandiosity, hostility, suspiciousness, hallucinatory behaviour, unusual thought content and excitement, was administered at pre-infusion, immediately post-infusion, 1 and 2 h post-infusion.

### Statistical analysis

Statistical analyses were carried out with the Statistical Package for Social Sciences (version 24, SPSS Inc., IBM, Armonk, NY, USA). Separate repeated measures analyses of variances (ANOVA) were used to assess changes in MMN indices of amplitude (µV), latency (ms), theta (4–8 Hz) EROs (µV) and theta PLF.

For frontal MMN amplitude, theta ERO and theta PLF analyses, within-group factors were drug condition (KET, ketamine; MID, midazolam), time (pre-infusion, immediately post-infusion, 2 h post-infusion) and electrode site (F3 (left), F4 (right), Fz (midline)). ANOVAs for mastoid (temporal) MMNs were similar except for the electrode site factor (TP9 (left) and TP10 (right)). N1 amplitude and latency, as well as MMN latency, did not contain a site factor as they were analysed at Fz only; latency for temporal sites included TP9 and TP10. Natural logged (ln) source-localised CSD (A/m²) for each ROI were analysed separately.

The main interaction effects of interest (a priori) for all measures were drug condition × time or drug × time × electrode (where both were significant) interactions and followed-up with Bonferroni adjusted pairwise comparisons. Partial eta-squared (ηp²) effect sizes are reported.

Relationships between MMN indices at baseline and their acute ketamine-induced changes (i.e. post-infusion – pre-infusion, Δpost-infusion and 2 h post-infusion – pre-infusion, Δ2 h post-infusion) and early and sustained absolute change in MADRS (early change scores (i.e. 2 h post-infusion – pre-infusion, Δ2 h; 1 day – pre-infusion, Δ1 d), sustained change scores (i.e. Post-Phase 2 – Baseline, ΔPh2; Post-Phase 3 – Baseline, ΔPh3)) using Pearson correlations. Correlations between MMN indices and acute changes in BPRS-P (ΔBPRS-P) symptoms were also performed.

Only electrophysiological variables (baseline and ketamine-induced changes) that were correlated with early and sustained change in MADRS were examined in terms of their predictive ability. Separate stepwise multiple regressions (Criteria: Probability-of-F-to-enter ⩽0.050, Probability-of-F-to-remove ⩾0.100) were performed with Δ2 h, Δ1 d, ΔPh2 and ΔPh3 for MADRS total score as dependent variables. As the early change in depressive symptoms can be predictive of later response, we also included early change in MADRS (i.e. Δ2 h and Δ1 d) in the regressions for sustained response (ΔPh2, ΔPh3).

## Results

### Demographics, clinical outcome and psychoactive symptom measures

Clinical outcome measures for the primary study outcomes are presented in Phillips et al. (2019). Twenty-four participants were included in the EEG portion of the study, and 23 completed both EEG sessions of Phase 1. One participant withdrew from the study during Phase 2 after receiving three infusions. None of the participants had mania, hypomania, post-traumatic stress disorder, alcohol or substance abuse or dependence, psychotic features, anorexia nervosa, bulimia nervosa or antisocial personality disorder. Demographic features and clinical characteristics of the sub-sample of participants are listed in Table 1.

**Table 1.** Demographic features and clinical characteristics of participants (*N* = 24). Means and standard deviations are presented.

Variable	Means (± SD), *N*s
Sex	14F/10M
Age (years)	41.7 ± 12.3
Weight (kg)	81.1 (17.7)
Body mass index (kg/m^2^)	27.1 (4.8)
Length of current episodes (years)	5.7 (4.4)
Major depressive episodes, single/recurrent	12/12
Failed antidepressant trials^a^, mean (SD)	3.1 (1.5)
Failed augmentation strategies^a^, mean (SD)	2.8 (1.0)
ECT nonresponder in current episode, *n* %	5, 21%
rTMS nonresponder in current episode, *n* %	1, 4%
Lifetime history of suicide attempt, *n* %	6, 25%
Comorbid panic disorder^b^, *n* %	2, 8%
Comorbid agoraphobia^b^, *n* %	6, 25%
Comorbid social phobia^b^, *n* %	6, 25%
Comorbid obsessive compulsive disorder^b^, *n* %	2, 8%
Comorbid generalised anxiety disorder^b^, *n* %	5, 21%

ECT: electroconvulsive therapy; F: female; M: male; MADRS: Montgomery-Åsberg depression rating scale (Montgomery and Asberg, 1979); rTMS: repetitive transcranial magnetic stimulation.

a Number of failed antidepressant trials and augmentations during current episode according to the Antidepressant Treatment History Form (Sackeim, 2001).

b Assessed with the Mini-International Neuropsychiatric Interview (Sheehan, 1998).

The antidepressant response rate (i.e. participants who had a ⩾50% decrease in their scores on the MADRS) to a single ketamine infusion at 2 h post-infusion was 16.7% (4 responders) and 25% (6 responders) at 24 h (1 day) post-infusion. The response rate for midazolam was 0% at all time points. The average number of days between the two sessions of Phase 1 was 9.7 (±4.6 S.D., range: 7–22 days). The response rate at the end of Phase 2 following repeated infusions was 57% (13 responders). Thirteen participants were entered into Phase 3, and 77% of those participants continued to meet treatment response criteria at the end of Phase 3 (Table 2).

**Table 2.** a. Montgomery-Åsberg depression rating scale (MADRS) means ± SD; b. Changes in brief psychiatric rating scale – positive symptoms (BPRS-P), means ± SD.

a.	Drug Session	Baseline	Pre infusion	2 h post	1 day post	1 week post	End of phase 2	End of phase 3
MADRS total score	KET	34.7 (4.1)	34.3 (4.5)	25.1 (8.5)	23.1 (8.8)	28.7 (9.3)	20.4 (13.1)	11.2 (6.7)
	MID		34.1 (5.3)	30.5 (6.4)	31.0 (5.3)	33.0 (6.6)		
Response	KET	–	–	16.7% (4 R/20 NR)	25.0% (6 R/18 NR)	8.3% (2 R/22 NR)	56.5% (13 R/ 10 NR)	76.9% (10 R/ 3 NR)
	MID		–	0.0% (24 NR)	0.0% (23 NR)	0.0% (23 NR)		
b.		Pre-infusion		Post-infusion	1 h post-infusion		2 h post-infusion	
BPRS-P score	KET	8.2 (0.4)		10.7 (3.5)	8.0 (0.0)		8.0 (0.0)	
	MID	8.0 (0.0)		8.0 (0.2)	8.0 (0.0)		8.0 (0.2)	

BPRS-P: brief psychiatric rating scale – positive symptoms; KET: ketamine; MADRS: Montgomery-Åsberg depression rating scale (Montgomery and Asberg, 1979); MID: midazolam; NR: non-responder; R: responder.

### Changes in MMN measures with ketamine and midazolam

**Figure 3.**
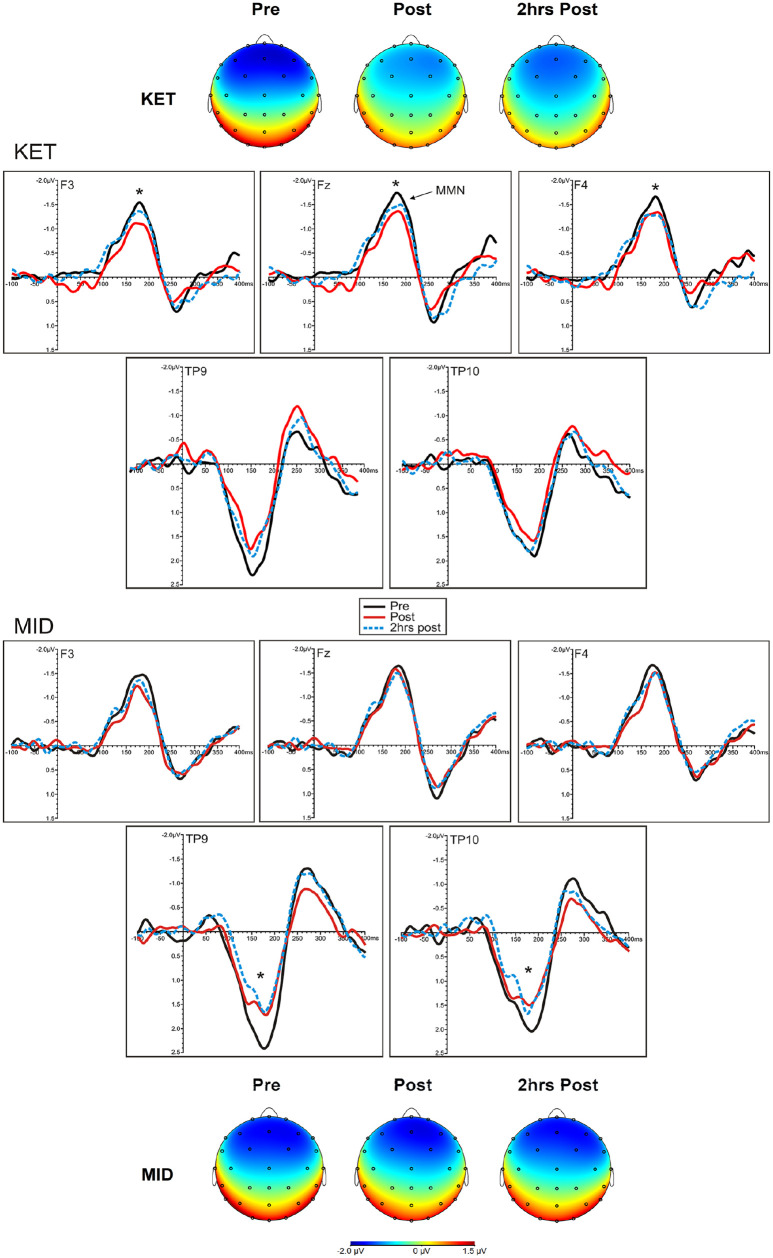
Grand averaged frontal (F3, left; Fz, midline; F4, right) and temporal (TP9, left; TP10, right) mismatch negativity (MMN) difference waveforms at each time point and drug condition. Peak MMN topographic maps at each time point and drug condition are displayed above and below each condition. *Time comparison, *p* < 0.05.

Frontal and temporal MMN amplitudes and latencies are shown in Table 3. Topographical headmaps at peak MMN latencies and grand average difference waveforms at the frontal electrode sites (F3, Fz, F4) and mastoid sites (TP9, TP10) during each drug condition (KET, MID) and recording time (pre, post, 2hrs post) are displayed in Figure 3.

**Table 3.** Mean (±SE) peak MMN frontal (F3, F4, Fz) and temporal (TP9, TP10) amplitudes (μV) and latencies (ms) at each time point and drug condition.

Drug Session	Time Point	Amplitudes (μV)					Latency (ms)		
		F3	F4	Fz	TP9	TP10	Fz	TP9	TP10
KET	Pre	−1.89 (.18)	−2.14 (.20)	−2.22 (.21)	2.61 (.29)	2.15 (.26)	187.9 (3.4)	160.4 (5.5)	164.0 (5.1)
	Post	−1.46 (.13)	−1.64 (.14)	−1.66 (.14)	2.04 (.27)	1.95 (.23)	190.2 (5.0)	165.2 (3.8)	168.3 (4.7)
	2 h Post	−1.68 (.15)	−1.61 (.17)	−1.78 (.18)	2.13 (.21)	2.04 (.18)	185.6 (3.9)	165.3 (4.1)	165.6 (4.8)
MID	Pre	−1.77 (.17)	−1.87 (.19)	−1.94 (.20)	2.75 (.26)	2.34 (.25)	186.8 (4.3)	163.3 (5.4)	164.9 (5.4)
	Post	−1.58 (.17)	−1.74 (.19)	−1.87 (.19)	1.96 (.24)	1.78 (.23)	186.2 (5.6)	160.0 (4.7)	157.4 (4.6)
	2 h Post	−1.61 (.19)	−1.79 (.18)	−1.82 (.19)	1.97 (.26)	2.02 (.19)	184.9 (5.1)	164.5 (4.9)	167.9 (4.7)

KET: ketamine; MID: midazolam; Post: immediately post-infusion; Pre: pre-infusion; 2 h post, 2 h post-infusion.

### Frontal amplitude and latency

rmANOVAs indicated a main interaction effect of drug × time × electrode *F*((4, 88) = 2.55, *p* = 0.04, ηp² = 0.10). MMN amplitudes were reduced from pre- to post-infusion (*p* = 0.007) and were larger at Fz compared to F3 (*p* = 0.0001). Within the drug × time × electrode interaction, MMN amplitudes were reduced from pre- to post-infusion for F3 (*p* = 0.003), F4 (*p* = 0.01) and Fz (*p* = 0.003), and from pre- to 2 h post-infusion for F4 (*p* = 0.03) and Fz (*p* = 0.04) in the ketamine condition (Figure S1, Supplemental file). No significant changes were observed in the midazolam condition.

No main interaction effects for drug × time (*F*(2, 44) = 0.16, *p* = 0.85, ηp² = 0.007) were observed for the analysis of frontal midline latency.

### Temporal amplitude and latency

rmANOVAs indicated main effects of time (*F*(2, 44) = 5.23, *p* = 0.009, ηp² = 0.19) and electrode (*F*(1, 22) = 5.57, *p* = 0.03, ηp² = 0.20), and a main interaction effect of time × electrode (*F*(2, 44) = 8.03, *p* = 0.001, ηp² = 0.27). Temporal MMN amplitudes were reduced from pre- to post-infusion (*p* = 0.03) and were larger at TP9 compared to TP10 (*p* = 0.03). Within the time x electrode interaction, amplitudes were larger at TP9 compared to TP10 at pre-infusion (*p* = 0.001) and were reduced immediately post- (*p* = 0.008) and 2 h-post infusion (*p* = 0.01) as compared to pre-infusion for TP9 only. Within a planned drug × time × electrode interaction (*F*(2, 44) = 0.46, *p* = 0.64, ηp² = 0.02), amplitudes were reduced from pre- to post-infusion for TP9 (*p* = 0.003) and TP10 (*p* = 0.005), and from pre- to 2 h-post-infusion (*p* = 0.0001) for TP9 only in the midazolam condition. No significant decreases were found in the ketamine drug condition.

No main interaction effects for drug × time (*F*(2, 44) = 0.81, *p* = 0.45, ηp² = 0.04) was observed for the analysis of temporal latency.

### Changes in N1 amplitude and latency with ketamine and midazolam

#### N1 amplitude

No main interaction effects for drug × time (*F*(2, 44) = 0.59, *p* = 0.56, ηp² = 0.03) were observed for the analysis of N1 amplitude.

#### N1 latency

A main interaction effects of drug × time (*F*(2, 44) = 3.46, *p* = 0.04, ηp² = 0.14) was found for N1 latency. Longer latencies were found at post-infusion as compared to pre- (*p* = 0.005) and 2 h-post-infusion (*p* = 0.01) within the midazolam condition only. N1 amplitudes and latencies are shown in Table 4. Grand average waveforms are shown in Figure S2.

**Table 4.** Mean (±SE) peak N1 amplitudes (μV) and latencies (ms) at each time point and drug condition.

Drug Session	Time Point	Amplitude (µV)	Latency (ms)
KET	Pre	−0.23 (.16)	104.0 (2.7)
	Post	−0.27 (.13)	104.9 (2.9)
	2 h post	−0.30 (.13)	104.7 (3.1)
MID	Pre	−0.18 (.14)	103.0 (2.5)
	Post	−0.20 (.14)	113.3 (3.4)
	2 h post	−0.24 (.14)	107.4 (2.8)

KET: ketamine; MID: midazolam; Post: immediately post-infusion; Pre: pre-infusion; 2 h post, 2 h post-infusion.

### Changes in theta ERO and PLF with ketamine and midazolam

rmANOVAs indicated a main interaction effect of drug × time (*F*(2, 44) = 3.47, *p* = 0.04, ηp² = 0.14). Theta ERO were reduced from pre- to post-infusion (*p* = 0.004) and 2 h post-infusion (*p* = 0.02), and ERO values were larger for Fz versus F3 (*p* = 0.0001). Within the drug × time interaction, ERO values were reduced from pre- to post-infusion (*p* = 0.006) and 2 h post-infusion (*p* = 0.01) in the ketamine drug condition only. No significant changes were observed in the midazolam condition.

No main interaction effects for drug × time (*F*(2, 44) = 0.58, *p* = 0.56, ηp² = 0.03) was observed for the analysis of theta PLF.

**Figure 4.**
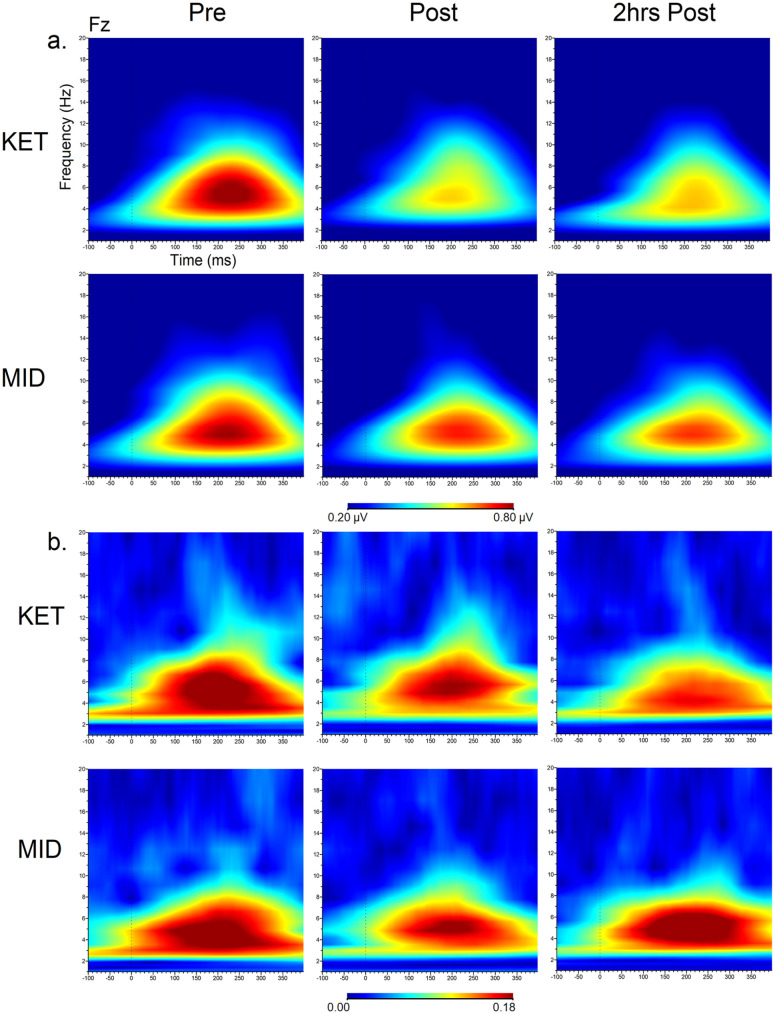
Grand averaged time frequency plots showing theta (a) event-related oscillations (µV) and (b) phase-locking factor at the frontal midline (Fz) site at each time point and drug condition.

Theta ERO and PLF time-frequency plots are shown in Figure 4, and values are shown in Table 5.

**Table 5.** Mean (±SE) peak MMN frontal event-related oscillations (μV) and phase locking factor (PLF) values at each time point and drug condition.

Drug Session	Time Point	ERO (μV)			PLF		
		F3	F4	Fz	F3	F4	Fz
KET	Pre	0.58 (0.06)	0.62 (0.06)	0.74 (0.08)	0.17 (0.02)	0.18 (0.02)	0.18 (0.02)
	Post	0.49 (0.06)	0.52 (0.06)	0.63 (0.09)	0.16 (0.01)	0.16 (0.02)	0.18 (0.02)
	2 h post	0.50 (0.06)	0.52 (0.05)	0.62 (0.07)	0.15 (0.02)	0.18 (0.02)	0.17 (0.02)
MID	Pre	0.58 (0.08)	0.60 (0.06)	0.71 (0.09)	0.17 (0.02)	0.18 (0.02)	0.18 (0.02)
	Post	0.49 (0.05)	0.49 (0.05)	0.61 (0.06)	0.14 (0.02)	0.15 (0.02)	0.15 (0.02)
	2 h post	0.48 (0.06)	0.52 (0.06)	0.60 (0.07)	0.16 (0.02)	0.16 (0.02)	0.17 (0.02)

KET: ketamine; ERO; event-related oscillations; MID: midazolam; PLF: phase-locking factor; Post: immediately post-infusion; Pre: pre-infusion; 2 h post, 2 h post-infusion.

### Changes in source-localised MMN peak ROIs with ketamine and midazolam

No main interaction effects for drug × time was observed for the three temporal generators (rSTL, lSTL, rITL). rmANOVAs indicated a main interaction effect of drug × time (*F*(2, 44) = 5.30, *p* = 0.009, ηp² = 0.19) for the ACC. CSD was reduced from pre- to post-infusion (*p* = 0.02) in the ketamine condition only. Ln-logged CSD values are shown in Table 6.

**Table 6.** Mean (±SE) ln-transformed source-localised CSD values (A/m^2^) of peak MMN generator activity.

Drug Session	Time Point	Peak MMN generator CSD			
		rSTL	lSTL	rITL	ACC
KET	Pre	−7.84 (0.22)	−9.54 (0.14)	−8.96 (0.13)	−9.26 (0.20)
	Post	−7.95 (0.23)	−9.34 (0.19)	−9.24 (0.18)	−9.80 (0.17)
	2 h post	−8.10 (0.23)	−9.40 (0.16)	−9.26 (0.19)	−9.77 (0.18)
MID	Pre	−8.00 (0.18)	−9.53 (0.16)	−9.14 (0.16)	−9.47 (0.18)
	Post	−8.06 (0.21)	−9.26 (0.13)	−9.23 (0.18)	−9.36 (0.17)
	2 h post	−8.22 (0.21)	−9.55 (0.18)	−9.46 (0.19)	−9.59 (0.20)

ACC: anterior cingulate cortex; CSD: current source density; KET: ketamine; lSTL: left superior temporal lobe; MID: midazolam; Post: immediately post-infusion; Pre: pre-infusion; 2 h post, 2 h post-infusion; rITL: right inferior temporal lobe; rSTL: right superior temporal lobe.

### Relationship between baseline and ketamine-induced changes in MMN with acute BPRS-P changes

Positive correlations between ΔBPRS and baseline theta Fz ERO (*r* = 0.44, *p* = 0.04, *N* = 23), and baseline F4 theta PLF (*r* = 0.44, *p* = 0.04, *N* = 23) were found.

### Relationship between baseline and ketamine-induced changes in MMN with MADRS change at Δ2 h, Δ1 d, ΔPh2 (*N* = 23) and ΔPh3 (*N* = 13)

**Figure 5.**
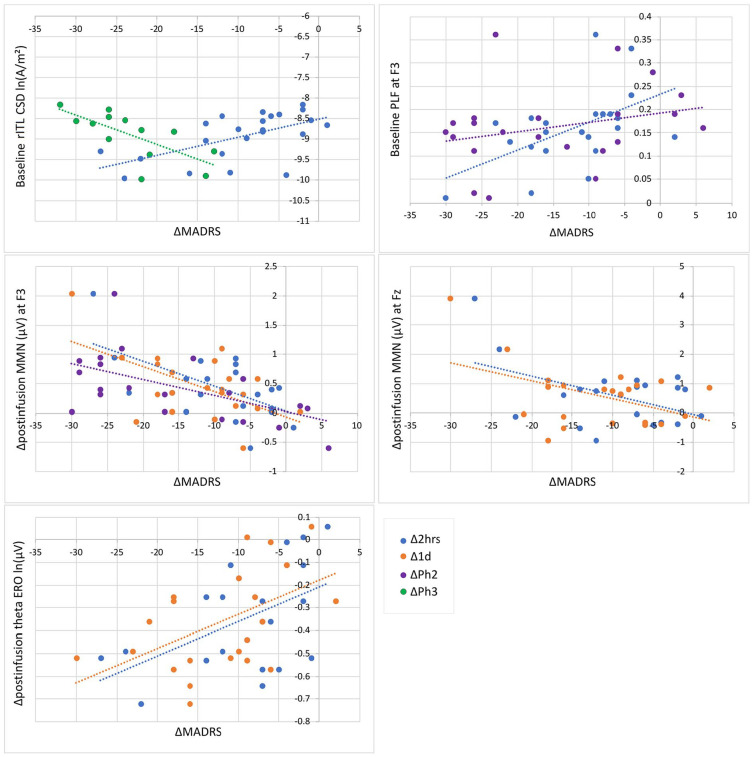
Scatterplots of significant correlations between baseline and ketamine-induced changes in mismatch negativity (MMN) measures with change in Montgomery-Åsberg Depression Rating Scale (MADRS) at 2 h (Δ2 h) and 1 day (Δ1 d) post-infusion, and change from baseline to end of phase 2 (ΔPh2) and end of phase 3 (ΔPh3). CSD: current source density; ERO: event-related oscillations; PLF: phase-locking factor.

Significant correlations are listed in Table 7, and scatterplots are displayed in Figure 5. Relationships were found between baseline and ketamine-induced MMN changes with both early and sustained treatment response. Early treatment response (at 2 h) was correlated with lower rITL peak CSD at baseline, and greater decreases in surface-level MMN measures (amplitude across all sites) immediately post-infusion as well as 2 h post-infusion (midline amplitude, theta ERO); these relationships continued to be observed with the clinical response 1 day post-infusion, in addition to a relationship showing less synchronised left phase-locking (F3 PLF) with greater decreases in MADRS that continued to be related to a sustained response (end of Phase 2). Decreases in left frontal MMN amplitude also continued to be related to larger MADRS decreases within Phase 2. Finally, higher baseline rITL peak CSD was related to sustained treatment response within repeated infusion responders (Phase 3).

**Table 7.** Significant correlations (*r, p*) between baseline MMN measures with change in Montgomery-Åsberg Depression Rating Scale (MADRS) at 2 h (Δ2 h) and 1 day (Δ1 d) post-infusion, and change from baseline to end of phase 2 (ΔPh2) and end of phase 3 (ΔPh3).

		MADRS			
MMN measure		Δ2 h (*N* = 23)	Δ1 d (*N* = 23)	ΔPh2 (*N* = 23)	ΔPh3 (*N* = 13)
Baseline	rITL CSD	*r* = 0.59, *p* = 0.003			*r* = −0.81, *p* = 0.001
	F3 PLF		*r* = 0.53, *p* = 0.009	*r* = 0.49, *p* = 0.02	
Δpost-infusion	F3 μV	*r* = −0.57, *p* = 0.005	*r* = −0.59, *p* = 0.003	*r* = −0.57, *p* = 0.006	
	F4 μV	*r* = −0.55, *p* = 0.007	*r* = −0.55, *p* = 0.007		
	Fz μV	*r* = −0.56, *p* = 0.006	*r* = −0.50, *p* = 0.02		
Δ2 h post	Fz μV	*r* = −0.47, *p* = 0.03	*r* = −0.45, *p* = 0.03		
	F4 μV		*r* = −0.44, *p* = 0.04		
	Theta ERO	*r* = 0.51, *p* = 0.01	*r* = 0.51, *p* = 0.01		

### MMN prediction of acute and sustained response to ketamine

Significant stepwise multiple regressions predicting change in MADRS at each time point were found. The model parameters (unstandardised Beta coefficient (*B*), standard error (SE), *t*-statistic (*t*) and significance (*p*)) for all regression analyses are reported in Table 8. With the addition of baseline and early change in MADRS, the only model that was changed was for Δ1 d MADRS (*F*(2, 22) = 14.25, *p* = 0.0001, *R*² = 0.59), which included Δ2 h MADRS (*B* = 0.59 ± 0.15, *t* = 3.84, *p* = 0.001) and F3 PLF baseline (*B* = 35.2 ± 13.5, *t* = 2.60, *p* = 0.02).

**Table 8.** Model parameters for regression analyses.

	Model	Independent variables	*B*	SE	*t*	*p*
Δ2 h	*F*(2, 22) = 8.31, *p* = 0.02, *R*² = 0.45	Δpost-infusion MMN (F3) µV	−6.13	2.30	−2.66	0.02
		Δpost-infusion ERO	12.67	5.77	2.20	0.04
Δ1 d	*F*(2, 22) = 11.58, *p* = 0.0001, *R*² = 0.54	Δpost-infusion MMN (F3) µV	−6.99	2.12	−3.30	0.004
		Baseline left PLF	40.60	14.04	2.89	0.009
ΔPh2	*F*(1, 21) = 6.17, *p* = 0.02, *R*² = 0.49	Baseline left PLF	67.93	27.36	2.48	0.02
ΔPh3	*F*(1, 12) = 21.58, *p* = 0.001, *R*² = 0.66	Baseline rITL CSD	−9.27	1.99	−4.65	0.001

B; beta coefficient; SE : standard error.

## Discussion

This ERP study examined acute changes in MMN-derived measures (amplitude, latency, ERO, PLF, peak MMN generator activity) with a sub-anesthetic dose of ketamine in comparison to the active placebo, midazolam. Baseline and ketamine-induced MMN changes were then examined in relation to early (Phase 1) and sustained (Phases 2 and 3) decreases in depressive symptoms with ketamine.

Within the randomised, double-blind, crossover phase of the study, decreases in MMN amplitude, theta ERO and the ACC frontal MMN generator were observed with ketamine, while midazolam decreased temporal MMN amplitudes and increased post-infusion N1 latency. These findings are in line with previous human studies that have observed acute decreases in MMN measures with ketamine (Rosburg and Kreitschmann-Andermahr, 2016), as well as decreases in the temporal MMN subcomponent and increases N1 latency with benzodiazepines (Rosburg et al., 2004). Decreases in theta ERO (Δpost-infusion and Δ2 h) and the frontal MMN generator (Δpost-infusion only) were also found in the ketamine condition, suggesting that the decrease in scalp-level frontal MMN amplitude values occur following decreases in the frontal generator and theta oscillations, as these measures have previously been found to underlie frontal MMN generation. There were no drug-induced changes in PLF, which may be due to the latency range extracted (based on frontal midline), as theta PLF measures have a stronger relation to the temporal subcomponent.

These reductions in MMN measures with ketamine indicate a brain that is less sensitive to its sensory context. As such, reduced MMN measures may be reflecting a decreased sensitivity to deviance, smaller prediction error signals, and could suggest weaker bottom-up prediction error signaling, potentially reflecting impairments in the brain’s ability to detect and process unexpected auditory events. Indexed by MMN, insensitivity to prediction error is thought to lead to and be propagated by depression (Badcock et al., 2017; Barrett et al., 2016) and may reflect NMDAR-dependent plasticity-mediated mechanisms (Schmidt et al., 2013; Weber et al., 2020).

Contrary to our hypothesis and to the findings of Sumner et al. (2020), we did not observe increases in MMN measures at our later recording time (2 h post-infusion). Major differences that could account for these findings are the time of recording, MMN task and deviant type, the participant sample and response rates. Notably, in Sumner et al.’s (2020) study, the MMN was recorded 3–4 h post-infusion, whereas our study recorded the MMN both immediately post-infusion and again at 2 h post-infusion. This timing difference suggests a potential pattern: MMN levels may initially decrease due to NMDAR antagonism but could then increase above baseline levels after some time, potentially aligning with Sumner et al.’s (2020) later recording window, though additional work is needed to elucidate this. The authors employed a roving auditory oddball task and a frequency (vs duration) deviant, which may capture different aspects of the MMN-indexed ketamine response. Additionally, the participants had less severe treatment resistance and exhibited much greater antidepressant responses at both 3 h and 1 day post as compared to our study. This may indicate a greater responsiveness for restoration of sensitivity to prediction errors impaired in MDD. With larger samples, comparisons could be made regarding depression severity levels and subsequent increases versus sustained decreases following sub-anesthetic ketamine infusions.

A greater increase in BPRS-P symptoms from pre- to immediately post-infusion was associated with higher baseline frontal midline theta ERO and right frontal PLF. In our previous related work in healthy controls (de la Salle et al., 2019), a similar relationship was found between an increase in derealization symptoms and baseline midline frontal PLF. These findings suggest that individuals with higher event-related theta oscillations and phase synchronisation are more susceptible to the ‘positive’ symptoms induced by ketamine. As theta ERO has been associated with passive attention and stimulus orientation (Karakaş, 2020), these individuals may be more sensitive to the psychotomimetic effects of ketamine.

Early decreases in depressive symptoms at 2 h and 1 day post-infusion were related to decreases in MMN amplitudes and theta ERO, as well as lower baseline rITL peak MMN generator activity (Δ2 h) and higher left frontal baseline PLF values (Δ1 d). Sustained decreases in depressive symptoms were related to decreases in left frontal MMN amplitude and lower left frontal baseline PLF (ΔPh2), and higher baseline rITL peak MMN generator activity (ΔPh3). These findings indicate that a better early response to a single sub-anesthetic ketamine administration is related to greater decreases in MMN measures (amplitude, theta ERO), with frontal left decreases (at F3) continuing to be related to larger MADRS decreases within Phase 2, in combination with less baseline left frontal PLF. By examining these relationships at different time points, we can attempt to discern who may respond positively both initially and in a sustained manner, and whether baseline measures versus ketamine-emergent change scores are equally or more informative. Indeed, several of these ketamine-induced changes were found to be predictive of decreases in early (left frontal MMN decreases in amplitude and theta ERO, baseline left PLF) and sustained (baseline left PLF, right inferior temporal activity) depressive symptoms and were able to explain 0.45–0.66 of the variance. Together, these findings suggest that early and sustained ketamine responders may be characterised by (1) a less synchronised phase-locking (left hemisphere) activity at baseline, (2) a more responsive NMDAR system as indexed by acute decreases in MMN amplitude and theta ERO and (3) a differential activation in the right inferior temporal lobe.

In Sumner et al. (2020), ketamine induced higher forward connectivity modulation from the right primary auditory cortex to the right inferior temporal cortex (for deviant stimuli), which was correlated with greater decreases in depressive symptoms. As well, ketamine increased the strength of activation of the inferior temporal cortex. In the current study, less peak activity in rITL was related to a change in depressive symptoms 2 h post-infusion, while more peak activity in this region was related to a change in depressive symptoms at the end of Phase 3. This can be explained when considering that most patients were non-responders at 2 h and most were responders at the end of Phase 3. As such, higher peak right interior temporal lobe activity was related to a greater antidepressant response.

### Limitations

Several limitations should be considered. There were no MMN recordings at 1 day post-infusion, which is when the greatest response to ketamine is observed, or during the sustained treatment phases to assess longer-term changes in MMN measures following repeated and maintenance ketamine infusions. There was no separate baseline session outside the testing (pre-infusion) days. Only one deviant was employed, therefore it cannot be determined whether differing patterns of ketamine-induced changes would have been observed with multiple deviant types. Additionally, the inherent spatial resolution limitations may have influenced the source-localised MMN generator values.

The participants in this study were limited to individuals enrolled in a larger clinical ketamine trial who were willing and able to participate in this electrophysiological component. Therefore, our sample was not large enough for a binomial analysis of responders versus non-responders and remitters versus non-remitters at each time point, and the Phase 3 analysis was limited due to the inclusion of treatment responders only. While midazolam is superior to saline as a placebo for ketamine, its lack of psychoactive side effects may have influenced the blind within the first phase of the study. The participants in this study had a high level of illness severity and treatment resistance, therefore the generalizability to other MDD populations may be limited. Finally, the fact that participants remained on their psychotropic medications throughout the trial limits the interpretability of the current findings, though these were stable dosages (>6 weeks) with no change throughout the trial.

Future studies would benefit from additional MMN recording time points which are both acute and later response times. This would allow for more detailed assessments of the potential transition from an acute decreased state indexing NMDAR antagonism, to an increase representing enhanced synaptic plasticity. Analyses of ketamine-induced event-related synchronisation/desynchronisation, connectivity between MMN generators and/or between the default mode network and the salience network, as well as measures of cross-frequency coupling and their relation to the rapid and sustained antidepressant response would also be valuable.

## Conclusion

To our knowledge, this is the first study to examine acute ketamine-induced MMN changes in relation to antidepressant efficacy with repeated ketamine infusions. Baseline and ketamine-induced decreases in multiple MMN-derived measures were related to and predictive of early and sustained decreases in depressive symptoms. Measures of MMN activity are thus feasible measures for investigating brain dynamics of NMDAR antagonism and synaptic plasticity resulting from sub-anesthetic, antidepressant doses of ketamine in TR MDD.

## Supplemental Material

sj-docx-1-jop-10.1177_02698811251319456 – Supplemental material for Acute subanesthetic ketamine-induced effects on the mismatch negativity and their relationship to early and sustained treatment response in major depressive disorder Supplemental material, sj-docx-1-jop-10.1177_02698811251319456 for Acute subanesthetic ketamine-induced effects on the mismatch negativity and their relationship to early and sustained treatment response in major depressive disorder by Sara de la Salle, Jennifer L Phillips, Pierre Blier and Verner Knott in Journal of Psychopharmacology
