# Supplementary material for: Acute subanesthetic ketamine-induced effects on the mismatch negativity and their relationship to early and sustained treatment response in major depressive disorder
Source: J Psychopharmacol. 2025 Feb 26;39(6):577–92. doi: 10.1177/02698811251319456 (PMC12205165; doi:10.1177/02698811251319456)
Supplement: sj-docx-1-jop-10.1177_02698811251319456 – Supplemental material for Acute subanesthetic ketamine-induced effects on the mismatch negativity and their relationship to early and sustained treatment response in major depressive disorder [file sj-docx-1-jop-10.1177_02698811251319456.docx]

**Supplemental Materials**


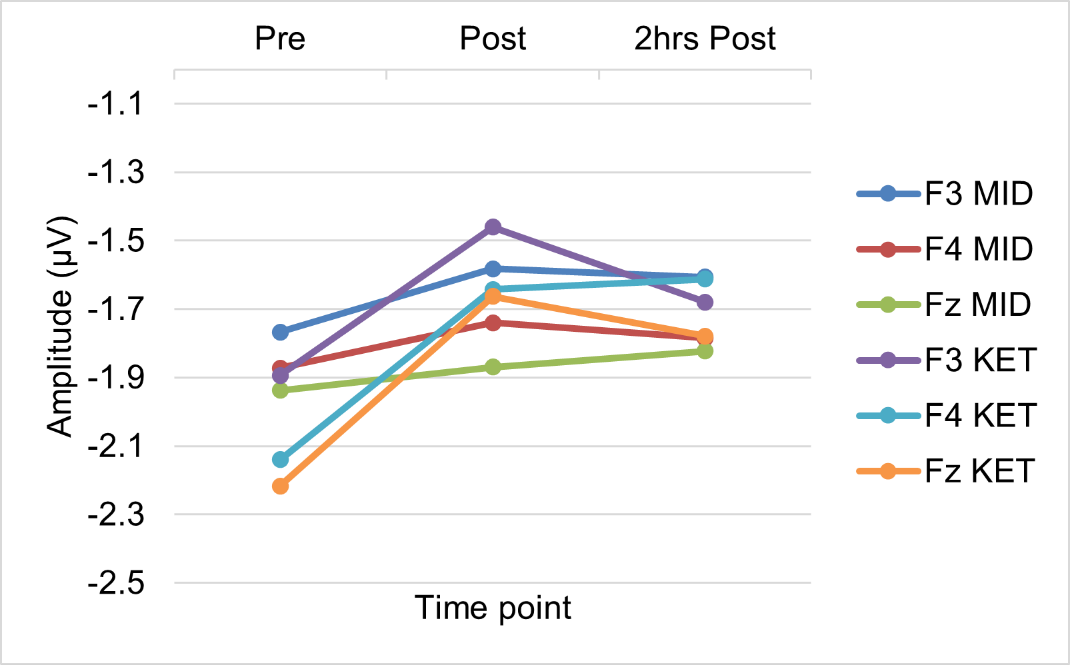


**Figure S1.** Mismatch negativity (MMN) amplitudes (µV) at frontal electrodes (F3, left; Fz, midline; F4, right) by time point and drug condition.


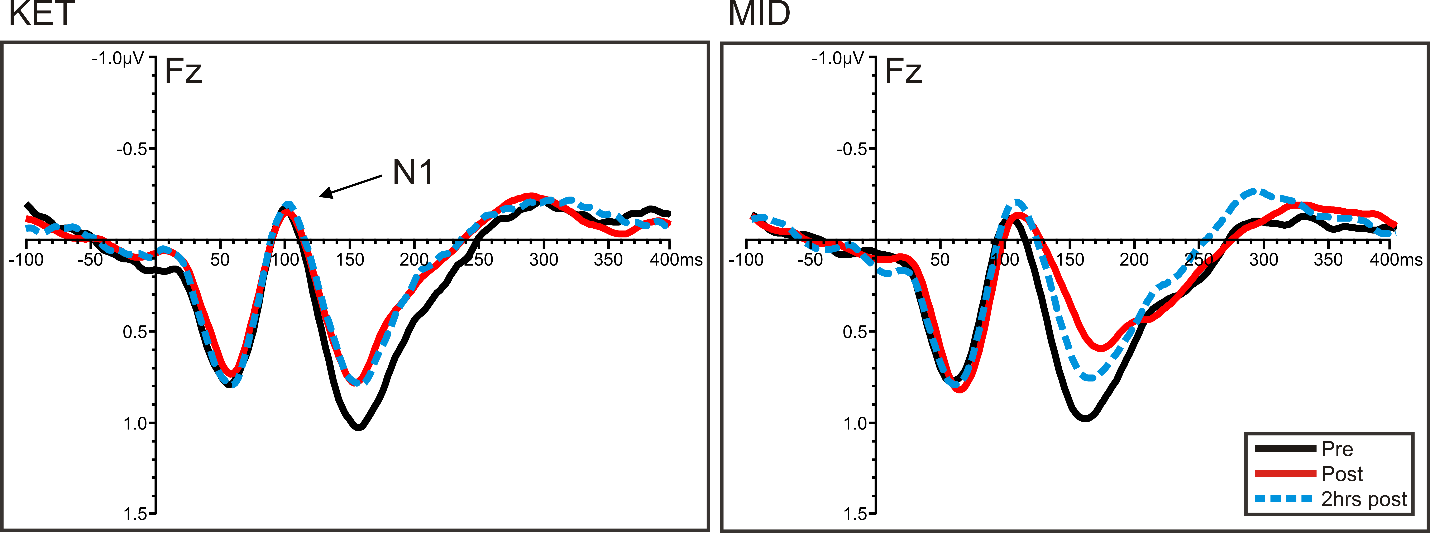


**Figure S2**. Frontal midline (Fz) grand averaged N1 waveforms at each time point and drug condition. *=time comparison, p<.05
